# Purine nucleoside phosphorylase inhibitors - an immunotherapy with novel mechanism of action for the treatment of melanoma

**DOI:** 10.1186/2051-1426-3-S2-P292

**Published:** 2015-11-04

**Authors:** Shanta Bantia

**Affiliations:** 1 Nitor Therapeutics, Birmingham, AL, USA

## Introduction

Contrary to expectations based on the immuno-compromised clinical phenotype of the PNP-deficient patients, the present study demonstrates that PNP inhibitors (PNPi) can activate immune cells that can help mount a robust antitumor response. PNP deficiency in humans lead to elevation of plasma guanosine [1]. In the present study, we demonstrate that guanosine can activate toll like receptor 2 (TLR2) and TLR4. Powerful immune-stimulatory properties of TLR2 and TLR4 agonist have been exploited for their potential as anti-cancer agents and as an adjuvant in cancer vaccines.

## Method

TLR stimulation is tested in-vitro by assessing NF-κB activation in HEK293 cells expressing a given TLR. In mouse melanoma model, cancer cells were injected subcutaneously and treatment with the NTR001 was initiated on day 6 after injection of tumor cells. Tumor volume and survival were recorded every 3-4 days. Mouse tetanus toxoid vaccine model was used to evaluate the adjuvant effect of NTR001.

## Results

Guanosine (100 uM) exhibits a significant stimulatory effect on human TLR2 and TLR4 (p < 0.0001 vs vehicle), alone and in combination with PNPi, NTR001 (10 uM). Guanosine demonstrates no effect on TLR3, TLR5, TLR7, TLR8 and TLR9. NTR001 as single agent demonstrates no effect on any of the TLRs. Treatment with NTR001 at doses 30 mg/kg every other week given p.o. and 5 mg/kg given every day in drinking water resulted in a significant decrease in tumor volume compared to the vehicle treated group (30 mg/kg and 5mg/kg p < 0.05 vs vehicle). Twenty percent of mice survived in the 5 mg/kg dose group whereas no mice survived in the vehicle and 30 mg/kg dose groups. The immune potentiating effect of NTR001 was further confirmed in mouse tetanus toxoid model where it demonstrated increase in both antibody titers and interferon-g levels.

## Conclusion

PNP inhibitors represent a novel approach, to enhance the immune system through activation of TLR2 and TLR4, for the treatment of melanoma and other malignancies. Combinations of NTR001 and/or guanosine with other cancer immunotherapies such as checkpoint modulators, CTLA-4 antagonist, PD-1 antagonist, and IDO-1 inhibitors will be explored further.

